# Supplementary material for: Pathogenic NLRP3 mutants form constitutively active inflammasomes resulting in immune-metabolic limitation of IL-1β production
Source: Nat Commun. 2024 Feb 6;15:1096. doi: 10.1038/s41467-024-44990-0 (PMC10847128; doi:10.1038/s41467-024-44990-0)
Supplement: Supplementary file 1 — Supplementary Information [file 41467_2024_44990_MOESM1_ESM.pdf]

Pathogenic NLRP3 mutants forms constitutively active inflammasomes resulting in immune-metabolic limitation of IL-1β production

Cristina Molina-López, Laura Hurtado-Navarro, Carlos J. García, Diego Angosto-Bazarra, Fernando Vallejo, Ana Tapia-Abellán, Joana R. Marques-Soares, Carmen Vargas, Segundo Bujan-Rivas, Francisco A. Tomás-Barberán, Juan I. Arostegui, Pablo Pelegrin

SUPPLEMENTARY FIGURES

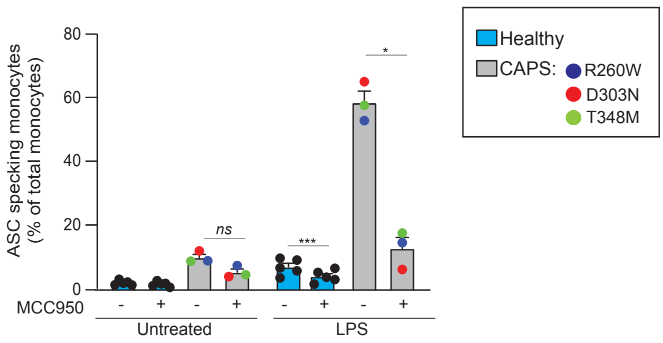

Supplementary Figure 1. Monocytes from CAPS patients show a constitutive activation of the NLRP3 inflammasome

Percentage of ASC specking monocytes from healthy donors (blue bars, *n*= 5) and CAPS patients (grey bars, p.R260W, p.D303N and p.T348M, *n*= 1 each variant, represented by a different color) after whole blood treated or not for 6 h with LPS (0.1 µg/ml), in the presence or absence of MCC950 (10 µM). *t*-test two-sided was performed to compare between MCC950 treated and untreated groups; significance levels are indicated as follows: *p < 0.05; ***p < 0.0002; *ns* indicates no significant difference (p > 0.05). Source data are provided as a Source Data file.

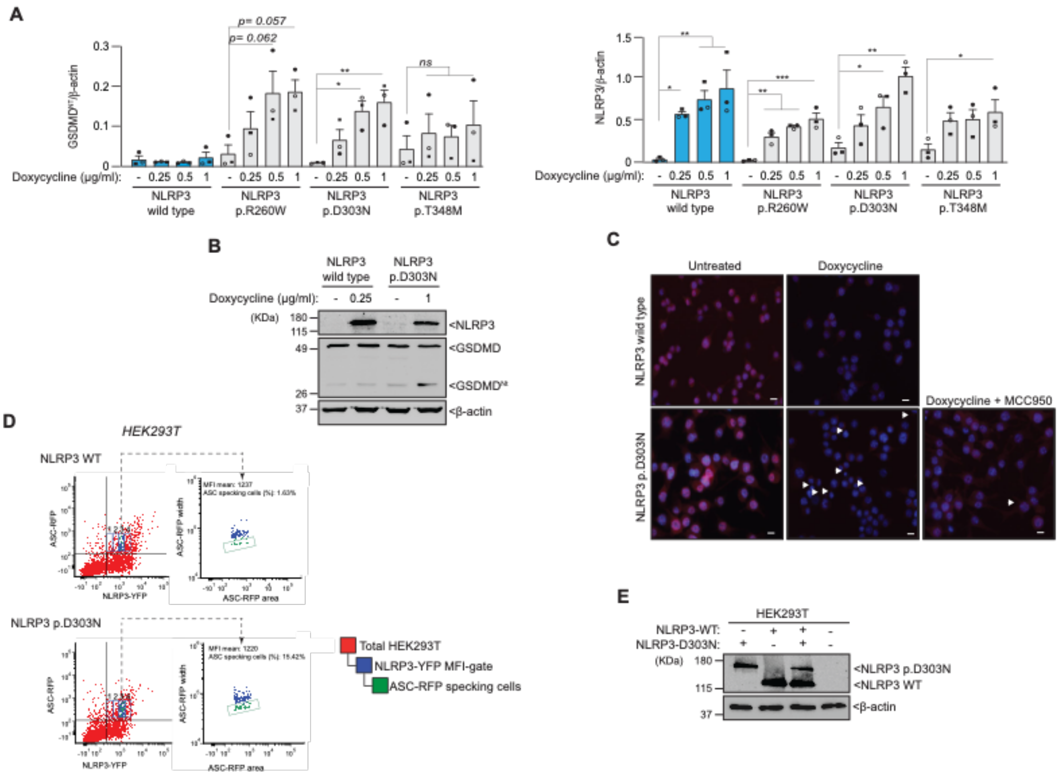

Supplementary Figure 2. Expression of CAPS-associated NLRP3 variants in macrophages results in a constitutive active inflammasome.

(**A**) Ratio of $GSDMD^{NT}$/β-actin (left) or NLRP3/β-actin (right) from Western blots as the ones presented in Figure 1A; *n*= 3 different Western blots corresponding to independent experiments.

(**B**) Western blot for NLRP3, GSDMD, and β-actin in cell lysates from *Nlrp3*$^{-/-}$ immortalized macrophages (iMos) treated for 16 h with or without doxycycline (0.25 or 1 µg/ml) to respectively induce the expression of the human wild type NLRP3 or the p.D303N variant.

(**C**) Representative fluorescence images of *Nlrp3*$^{-/-}$ iMos as the ones quantified in Figure 1C; ASC is shown in red, DAPI is shown in blue; scale bar=10 µm; arrowheads denote ASC specks. Images are representative of *n*= 3 independent experiments.

(**D**) Gating strategy to analyse the percentage of ASC specking cells in four different gates with increased expression of NLRP3-YFP wild type (WT, top) or p.D303N (bottom) calculated as mean fluorescence intensity (MFI). As example, the percentage of ASC specking cells is shown in the gate number three for NLRP3 expression.

(**E**) Western blot for NLRP3 and β-actin in cell lysates from HEK293T transfected with empty vector, NLRP3 wild type (WT), NLRP3 p.D303N-YFP or co-transfected with NLRP3 WT and NLRP3 p.D303N-YFP.

Western blots are representative of *n*= 3 independent experiments each. Graphics are representative of *n*= 3 independent experiments (each one represented by a different symbol) and data is represented as mean ± SEM; Ordinary one-way ANOVA test was used in panel A; significance levels are indicated as follows: * p < 0.05; **p < 0.0021; *ns* indicates no significant difference (p > 0.05). Source data are provided as a Source Data file.

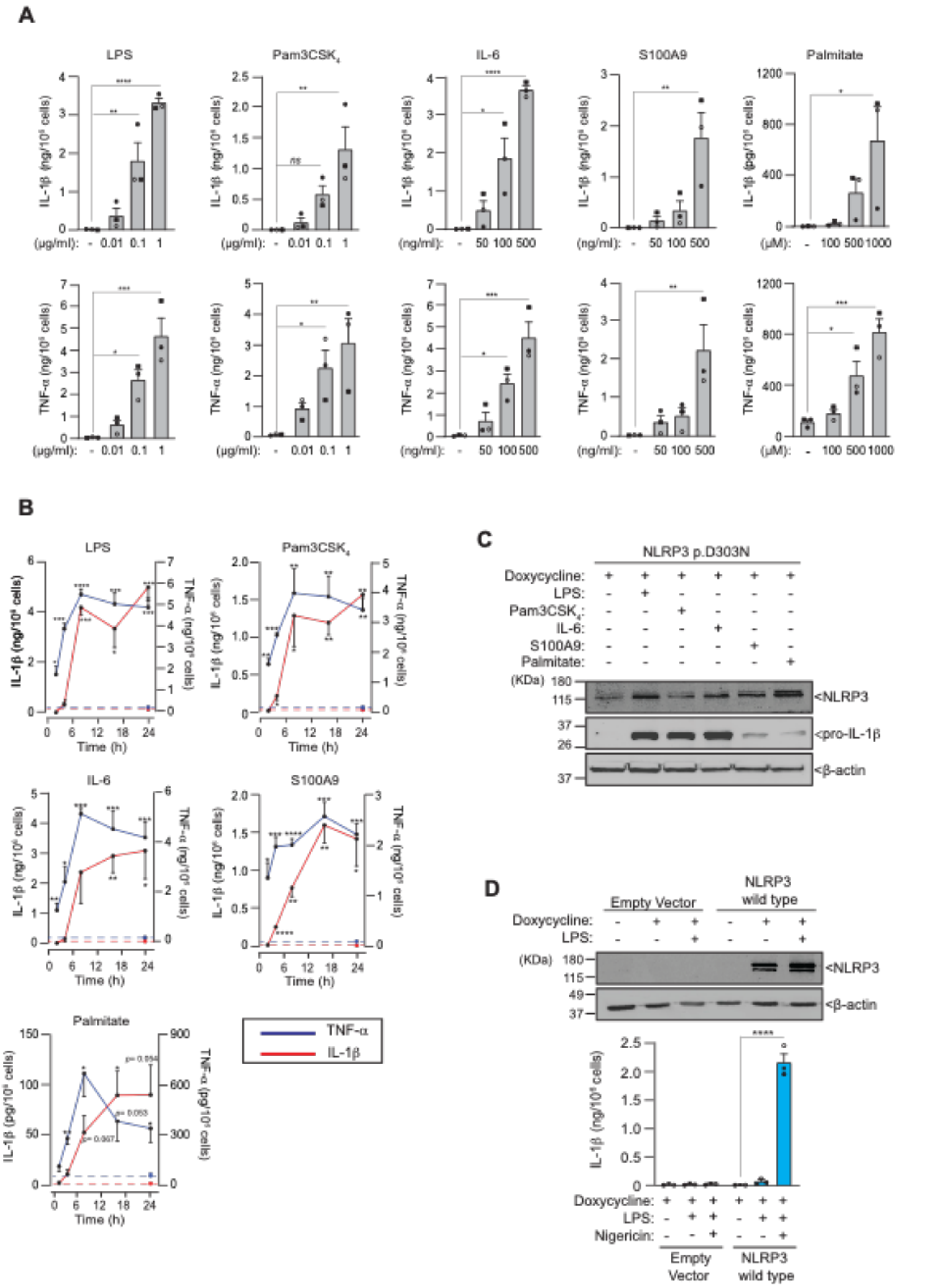

Supplementary Figure 3. NF-κB induction induces IL-1β release from macrophages expressing CAPS-associated NLRP3 variants.
(**A**) ELISA for IL-1β (top) and TNF-α (bottom) release from *Nlrp3*$^{-/-}$ immortalized macrophages (iMos) expressing the human NLRP3 p.D303N mutant induced after 16 h treatment with doxycycline (1 μg/ml) and different concentrations (as annotated) of LPS, Pam3-CSK4, recombinant IL-6, recombinant S100A9 or palmitate.

(**B**) ELISA for IL-1β (red line) and TNF-α (blue line) release from *Nlrp3*$^{-/-}$ iMos treated as indicated in A but for different times (0, 2, 4, 8, 16 and 24 h). The concentrations used were LPS 0.1 μg/ml, Pam3CSK$_4$ 1 μg/ml, recombinant IL-6 0.5 μg/ml, recombinant S100A9 0.5 μg/ml, and palmitate 1 mM. Dotted lines indicate basal cytokine release of IL-1β (red line) or TNF-α (blue line) in untreated iMos cultured for 24h.
(**C**) Western blot for NLRP3, IL-1β and β-actin in cell lysates from *Nlrp3*$^{-/-}$ iMos expressing the human NLRP3 p.D303N mutant induced after 16 h treatment with doxycycline (1 μg/ml) and palmitate (1 mM), recombinant S100A9 (0.5 μg/ml), recombinant IL-6 (0.5 μg/ml), LPS (0.1 μg/ml) or Pam3-CSK$_4$ (1 μg/ml).
(**D**) ELISA for IL-1β release or Western blot for NLRP3 and β-actin in cell lysates from *Nlrp3*$^{-/-}$ iMos transduced with an empty vector or a vector expressing the wild type NLRP3 after 16 h with or without doxycycline (1 μg/ml) and LPS (100 ng/ml), and then for the ELISA treated for additional 30 min with nigericin (10 μM) as indicated.
Western blots are representative of *n*= 3 independent experiments; Graphics average *n*= 3 independent experiments (each one represented by a different symbol) and data is represented as mean ± SEM; Ordinary one-way ANOVA test was used for panels A,D and *t*-test two-sided in panel B comparing each time with their respective untreated control; significance levels are indicated as follows: *p < 0.05; **p < 0.0021; ***p < 0.0002; ****p < 0.0001; *ns* indicates no significant difference (p > 0.05). Source data are provided as a Source Data file.

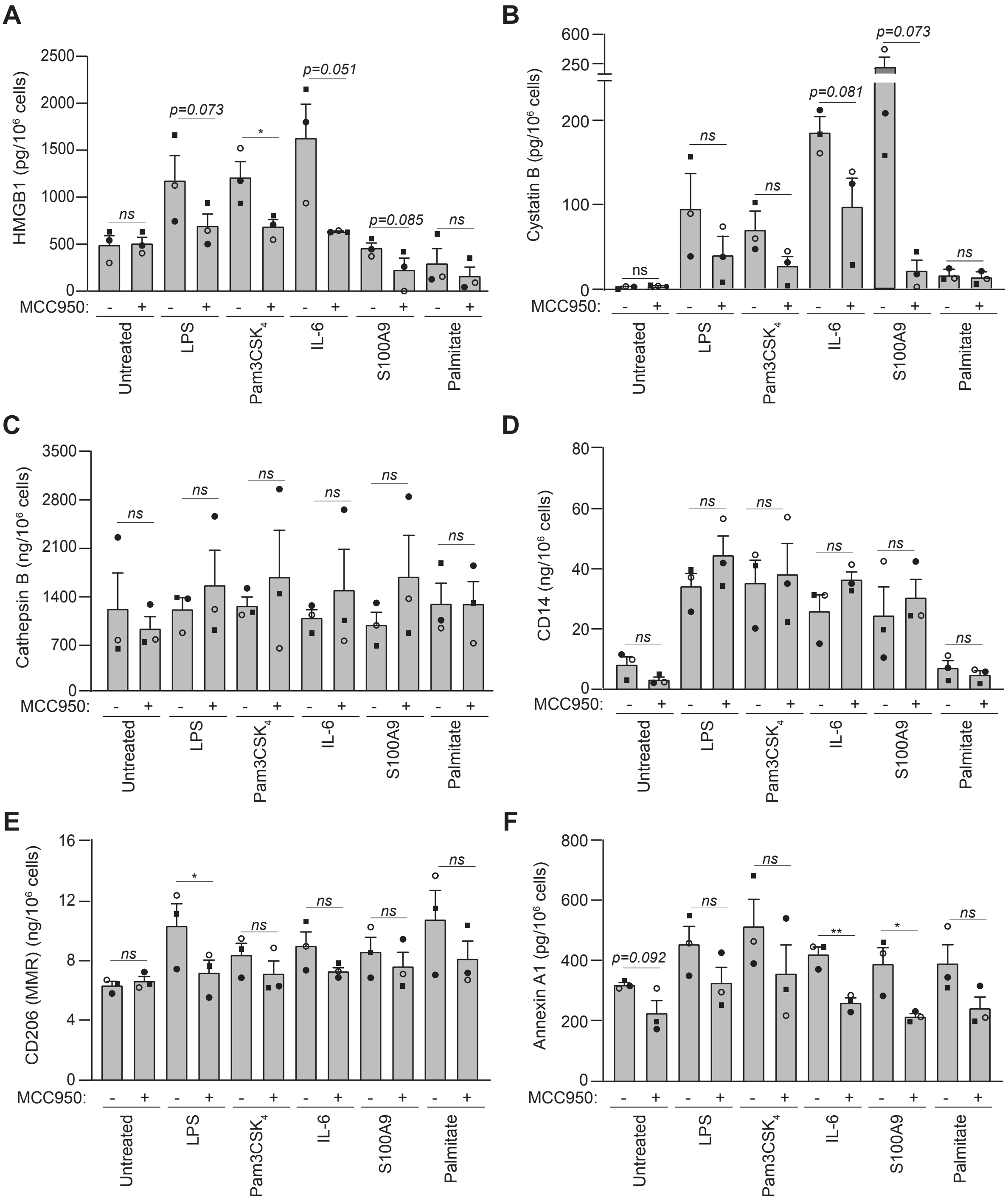

Supplementary Figure 4. Secretome of CAPS-associated NLRP3 variants.
(**A-F**) ELISA for the release of HMGB1 (A), cystatin B (B), cathepsin B (C), soluble CD14 (D), soluble CD206 (E) or annexin A1 (F) from *Nlrp3*$^{-/-}$ immortalized macrophages expressing the human NLRP3 p.D303N variant induced after 16 h treatment with doxycycline (1 µg/ml) and palmitate (1 mM), recombinant S100A9 (0.5 µg/ml), recombinant IL-6 (0.5 µg/ml), LPS (0.1 µg/ml) or Pam3-CSK_4 (1 µg/ml), in the absence or presence of MCC950 (10 µM).
Graphics are representative of *n*= 3 independent experiments (each one represented by a different symbol) and data is represented as mean ± SEM; *t*-test two-sided was used to compare the effect of MCC950 for each NF-κB inducer; significance levels are indicated as follows: *p < 0.05;**p < 0.0021; *ns* indicates no significant difference (p > 0.05). Source data are provided as a Source Data file.

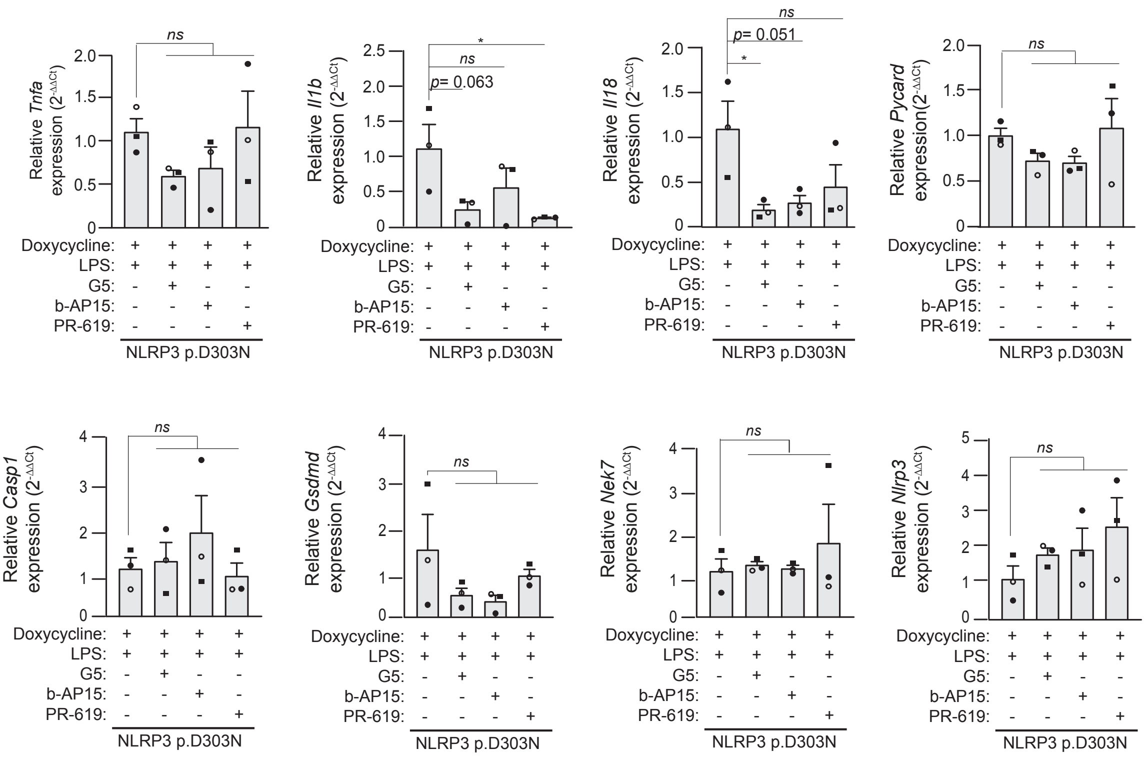

Supplementary Figure 5. Deubiquitinases inhibitors affect the expression of *Il1b*, *Il18*, but not the expression of other inflammasome components or *Tnfa*.
Gene expression ($2^{-\Delta\Delta Ct}$) relative to doxycycline and LPS treatment for *Tnfa*, *Il1b*, *Il18*, *Pycard*, *Casp1*, *Gsdmd*, *Nek7* and *Nlrp3* from *Nlrp3*$^{-/-}$ immortalized macrophages expressing the human NLRP3 p.D303N mutant induced after 16 h treatment with doxycycline (1 µg/ml) and then treated for 6 h with LPS (100 ng/ml), with or without G5 (5 µM), b-AP15 (5 µM) or PR-619 (10 µM).
Graphics are representative of *n*= 3 independent experiments (each one represented by a different symbol) and data is represented as mean ± SEM; Ordinary one-way ANOVA test was used; significance levels are indicated as follows: *p < 0.05; *ns* indicates no significant difference (p > 0.05). Source data are provided as a Source Data file.

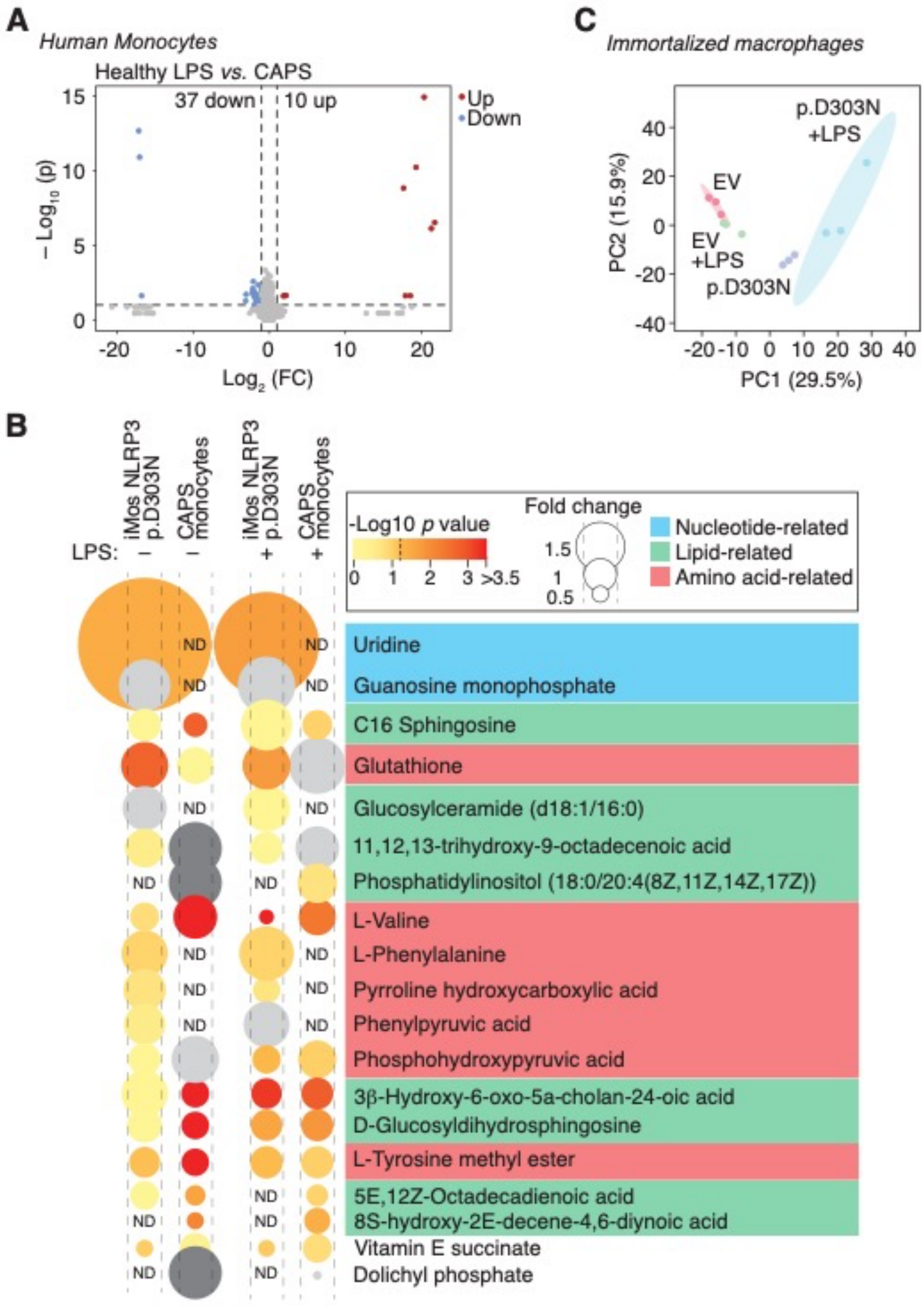

Supplementary Figure 6. Immunometabolism of myeloid cells expressing CAPS-associated NLRP3 variants

(**A**) Volcano plot of metabolites present in blood monocytes from healthy subjects (*n*= 4) and CAPS patients (*n*= 4), with expression (log_2 values) plotted against the adjusted P value for the difference in metabolite abundance; *t*-test two-sided. Metabolites that are significantly upregulated (red) or downregulated (blue) by one-fold or more in monocytes from CAPS patients are compared to those in monocytes from healthy subjects treated for 2 h with LPS (500 ng/ml).

(**B**) Abundance profile of metabolites tentatively identified in human CAPS monocytes (*n*= 4) and iMos expressing human NLPR3 p.D303N (*n*= 3) after treatment with doxycycline, both with or without LPS as in panels A and C respectively. Metabolites were grouped based on their properties: nucleotide-related (blue), lipid-related (green)

and amino acids-related (pink). The data are represented as circles, where the size indicates the fold change relative to healthy donor monocytes or iMos without doxycycline treatment, and the colour represents the $-\text{Log}_{10}$ *p*-value (the dotted line in the colour scale represents $p = 0.05$; *t*-test two-sided). Light grey indicates insufficient power to calculate statistics, while dark grey indicates that the metabolite was not detected in heathy donor monocytes and only in CAPS samples. ND denotes not detected.
(**C**) Principal component analysis (PCA) model of metabolomic profiles of *Nlrp3*$^{-/-}$ immortalized macrophages (iMos) expressing human NLRP3 p.D303N or not (empty vector, EV), treated with doxycycline (1 µg/ml) for 16 hours and then for 4 h with or without LPS (100 ng/ml) ($n = 3$). Source data are provided as a Source Data file.

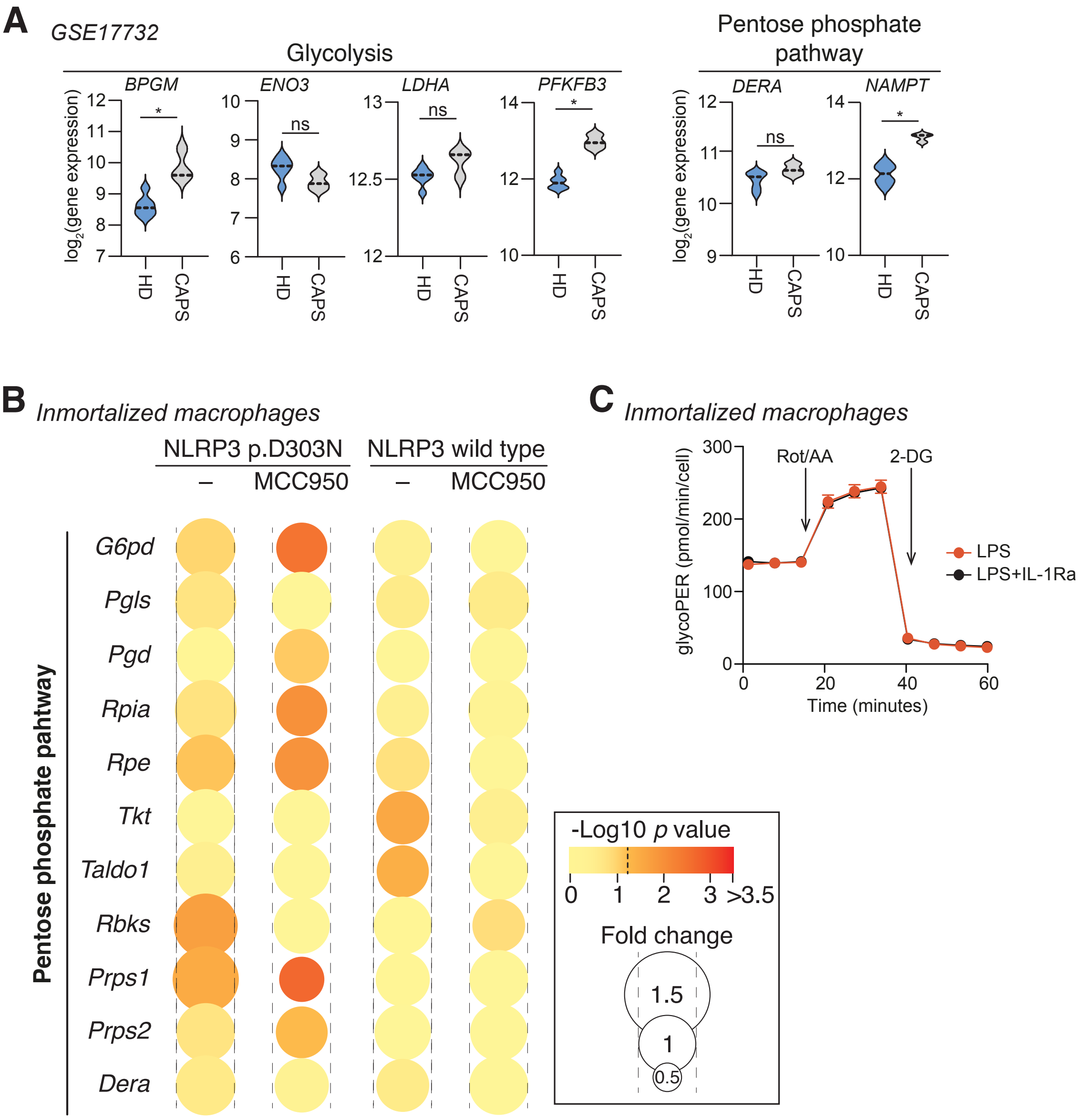

Supplementary Figure 7. Comparative analysis of gene expression in cells with CAPS-associated NLRP3 variants

(**A**) Glycolysis and pentose phosphate pathway gene expression in blood cells from healthy donors (HD, *n*= 6) and CAPS patients during active disease (*n*= 3) was represented as violin plot (using dataset GSE17732). The median is represented by the middle dotted line. Data were analysed using a *t*-test two-sided with Benjamini–Hochberg correction to compare gene expression. * denotes *p*< 0.05, while *ns* indicates no significant difference (*p*> 0.05).

(**B**) Relative expression of pentose phosphate pathway genes in *Nlrp3*$^{-/-}$ immortalized macrophages (iMos) treated for 16 h with or without doxycycline (1 µg/ml) to induce the expression of the human NLRP3 wild type or the p.D303N variant, in the absence or presence of MCC950 (10 µM). The data are represented as circles, where the size indicates the fold change of doxycycline treated cells vs untreated cells, or the fold change of doxycycline with MCC950 treatment vs doxycycline, and the colour represents the –Log_{10} *p*-value (the dotted line in the colour scale represents *p*= 0.05); *t*-test two-sided. The graphics represent data from three to four independent experiments.

(**C**) Seahorse analysis of glycolysis in *Nlrp3*$^{-/-}$ iMos treated for 16 h with doxycycline (1 µg/ml) to induce the expression of the human NLRP3 p.D303N variant and LPS (100 ng/ml), in the absence or presence of IL-1Ra (100 ng/ml). The data are derived from 4 biological replicates, which are representative of 2 independent experiments. Source data are provided as a Source Data file.

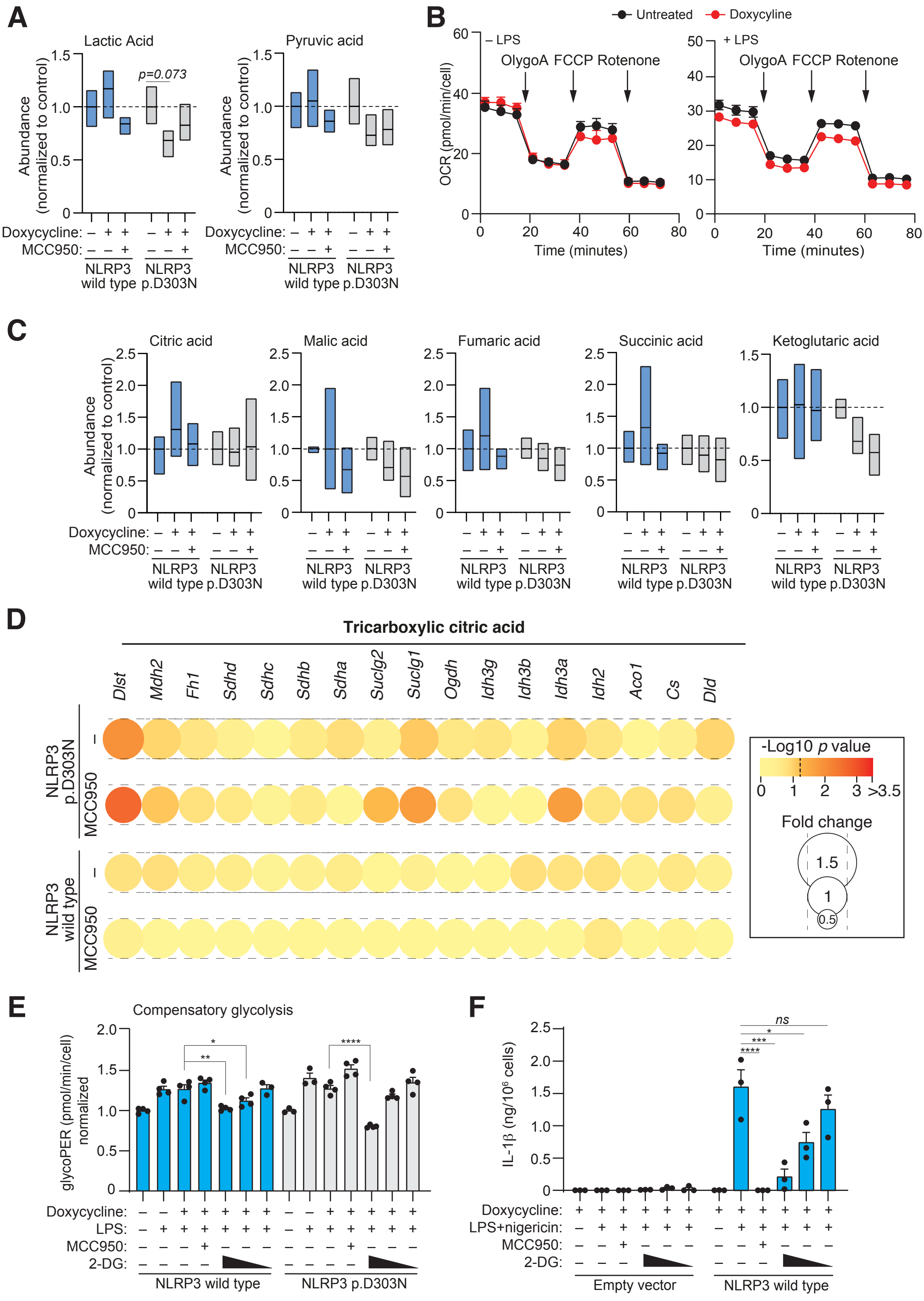

Supplementary Figure 8. Metabolic reprogramming in macrophages expressing the NLRP3 p.D303N variant

(**A**) Normalized abundance of lactic acid and pyruvic acid in *Nlrp3*$^{-/-}$ immortalized macrophages (iMos) expressing either the wild type NLRP3 or the p.D303N variant,

treated for 16 h with or without doxycycline (1 µg/ml) and MCC950 (10 µM). The graphics represent the data from *n*= 3 independent experiments, with the median indicated by the middle line. The dotted line represents the abundance level in control cells.

(**B**) Seahorse analysis of mitochondria oxygen consumption rate (OCR) in iMos treated for 16 h with or without doxycycline (1 µg/ml), in the absence (left) or presence (right) of LPS (100 ng/ml). The data represent 8 biological replicates, and is representative from three independent experiments.

(**C**) Normalized abundance of various metabolites of the tricarboxylic citric acid (TCA) cycle in iMos treated as described in panel A. The graphics are representative of three independent experiments, with the median indicated by the middle line. The dotted line represents the abundance level in control cells.

(**D**) Relative expression of TCA genes from iMos treated as described in panel A. The data are represented as circles, where the size indicates the fold change of doxycycline treated cells vs untreated cells, or the fold change of doxycycline with MCC950 treatment vs doxycycline, and the colour represents the $-\text{Log}_{10}$ *p*-value (the dotted line in the colour scale represents *p*= 0.05). The graphics represent data from three to four independent experiments.

(**E**) Compensatory glycolysis of iMos treated as described in panel A, but for 4 h and with 2-deoxy-D-glucose (2-DG, at different concentrations 0.1, 0.5 and 1 mM). The data represent 3-4 biological replicates, and is representative from two independent experiments.

(**F**) ELISA for IL-1β release from iMos expressing or not wild type NLRP3 treated for 4 h with doxycycline (1 µg/ml), MCC950 (10 µM), LPS (100 ng/ml) or 2-DG (0.1, 0.5 and 1 mM), and then treated for 30 min with nigericin (10 µM) (*n*= 3).

For panels B,E,F data are represented as mean ± SEM, for panels A,C data are represented as mean (middle line) and bounds of box represent the 25th to 75th percentile respectively; Ordinary one-way ANOVA test was used for panel E,F, and *t*-test two-sided was used for panels A,D; *p < 0.05; **p < 0.0021; ***p < 0.0002; *ns*, no significant difference (p > 0.05). Source data are provided as a Source Data file.

SUPPLEMENTARY TABLES

Supplementary Table 1. Demographic and clinical information of the individuals enrolled in this study.

	**Healthy controls**	**CAPS**
**N**	9	7
**Age** in years, mean (range) ± SD	32.89 (23-44) ± 8.13	51.85 (20-73) ± 17.45
p value		*p*= 0.011
**Gender**, N (%)		
Male	3 (66.6)	5 (71.4)
Female	6 (33.3)	2 (28.6)
p value		*p*= 0.131^{ns}
**Clinical data** *(only for CAPS patients)*		
**NLRP3 variant**, N (%)		
p.D303N		1 (14.3)
p.R260W		1 (14.3)
p.T348M		1 (14.3)
p.A439T		4 (57.1)
**Clinical phenotype**, N (%)		
MWS		6 (85.7)
MWS/CINCA		1 (14.3)
**Treatment**, N (%)		
Canakinumab		3 (42.9)
Anakinra		4 (57.1)

CINCA: chronic infantile neurologic cutaneous articular syndrome; MWS: Muckle–Wells syndrome; *ns*, no significant difference (p> 0.05); SD, standard deviation

Chi-square (χ^2) test was used for gender and *t*-test two-sided was used for age.

Supplementary Table 2. Tentative identification of metabolites in human monocytes that are differentially present in CAPS monocytes.

Metabolite	Formula	Mass	Polarity	error (ppm)	Fold change untreated	Fold change LPS
Glutathione	$C_{10}H_{17}N_3O_6S$	307.084	Pos	0.63	1.04	1.60
11,12,13-trihydroxy-9-octadecenoic acid	$C_{18}H_{34}O_5$	330.2406	Neg	-1.73	ND in untreated HD monocytes, only in CAPS	1.35
Phosphatidylinositol (18:0/20:4(8Z,11Z,14Z,17Z))	$C_{47}H_{83}O_{13}P$	886.5573	Neg	-0.43	ND in untreated HD monocytes, only in CAPS	1.22
L-Valine	$C_5H_{11}NO_2$	117.0789	Pos	4.05	1.34* *p*= 0.0002	1.14* *p*= 0.004
Phosphohydroxypyruvic acid	$C_3H_5O_7P$	183.9783	Pos	8.52	1.45	1.16
Vitamin E succinate	$C_{33}H_{54}O_5$	530.3978	Pos	7.5	0.96	0.89
3β-Hydroxy-6-oxo-5a-cholan-24-oic acid	$C_{24}H_{38}O_4$	390.2768	Pos	6.5	0.86* p= 5.58^10^{-5}	0.91* *p*= 0.001
D-Glucosyldihydrosphingosine	$C_{24}H_{49}NO_7$	463.3509	Pos	1.18	0.85* *p*= 0.0001	0.91* *p*= 0.013
L-Tyrosine methyl ester	$C_{10}H_{13}NO_3$	195.0899	Neg	-0.68	0.85* p= 1.13^10^{-7}	0.92
Butyl ethyl malonate	$C_9H_{16}O_4$	188.1049	Neg	-2.68	0.82* *p*= 0.0005	0.94
C16 Sphingosine	$C_{16}H_{33}NO_2$	271.2502	Pos	-1.41	0.74* *p*= 0.001	0.92
5E,12Z-Octadecadienoic acid	$C_{18}H_{32}O_2$	280.2403	Pos	2.21	0.63* *p*= 0.021	0.67
8S-hydroxy-2E-Decene-4,6-diynoic acid	$C_{10}H_{10}O_3$	178.0631	Pos	3.7	0.53* *p*= 0.0079	0.81* *p*= 0.034
Dolichyl phosphate	$C_{25}H_{45}O_4P$	440.306	Pos	4.5	ND in untreated HD monocytes, only in CAPS	0.27

Abbreviations: HD: Healthy donor; ND: Non-detected; Neg: negative; Pos: positive; *t*-test two-sided *p<0.05.

Supplementary Table 3. Tentative identification of metabolites in immortalized mouse macrophages that are differentially present when NLRP3 p.D303N is expressed.

Metabolite	Formula	Mass	Polarity	error (ppm)	Fold change untreated	Fold change LPS
Uridine	$C_9H_{12}N_2O_6$	244.0697	Neg	0.67	4.09* *p*= 0.03	3.22* *p*= 0.017
Guanosine monophosphate	$C_{10}H_{14}N_5O_8P$	363.0575	Neg	-1.37	1.59	1.76
C16 Sphingosine	$C_{16}H_{33}NO_2$	271.2508	Pos	-1.21	1.01	1.57
Glutathione	$C_{10}H_{17}N_3O_6S$	307.0837	Neg	0.31	1.45* *p*= 0.002	1.47* *p*= 0.016
Glucosylceramide (d18:1/16:0)	$C_{40}H_{77}NO_8$	699.5668	Neg	-2.69	1.24	1.43
11,12,13-trihydroxy-9-octadecenoic acid	$C_{18}H_{34}O_5$	330.241	Neg	1.14	1.10	0.99
L-Valine	$C_5H_{11}NO_2$	117.0789	Pos	-0,67	0.89	0.46* *p*= $3.35^\wedge10^{-5}$
L-Phenylalanine	$C_9H_{11}NO_2$	165.0787	Pos	-1.69	1.42	1.54
Pyrroline hydroxycarboxylic acid	$C_5H_7NO_3$	129.0424	Neg	-1.5	1.35	0.84
Phenylpyruvic acid	$C_9H_8O_3$	164.0488	Pos	8.87	1.26	1.29
Phosphohydroxypyruvic acid	$C_3H_5O_7P$	183.9784	Pos	6.04	0.96	0.87* *p*= 0.046
3β-Hydroxy-6-oxo-5a-cholan-24-oic acid	$C_{24}H_{38}O_4$	390.2773	Pos	0.74	1.32	0.87* *p*= 0.0004
D-Glucosyldihydrosphingosine	$C_{24}H_{49}NO_7$	463.3513	Pos	0.86	0.99	0.89
L-Tyrosine methyl ester	$C_{10}H_{13}NO_3$	195.0895	Neg	-0.22	0.95	0.92
Butyl ethyl malonate	$C_9H_{16}O_4$	188.1051	Neg	1.28	0.94	0.95
5E,12Z-Octadecadienoic acid	$C_{18}H_{32}O_2$	280.241	Pos	2.75	0.89	ND in LPS-macrophages NLRP3 p.D303N
Vitamin E succinate	$C_{33}H_{54}O_5$	530.3963	Pos	-1.56	0.53	0.52

Abbreviations: ND: Not-detected; Neg: negative; Pos: positive; *t*-test two-sided *p<0.05.

Supplementary Table 4. Biological processes enriched with upregulated genes upon NLRP3 p.D303N expression.

GO term	Gene count	*p*-value*	Benjamini–Hochberg**	Genes
Inflammatory response (GO:0006954)	30	1.86E-04	0.00944575	*Il1rn, Ddx3x, Dhx9, C5ar1, Hmgb1, Ptgs2, Cxcl2, Tnf, Ccl7, Zc3h12a, Nfkbiz, Ccl2, Ccr7, Cd14, Tnfrsf4, Map2k3, Ccl22, Stard7, Acod1, Ppbp, Tnfrsf1b, Nfkb1, Il17ra, Nfkb2, Cxcl10,*
Positive regulation of apoptotic process (GO:0043065)	30	1.32E-04	0.007331328	*Top2a, Tomm40, Ddx3x, Hmgb1, Ptgs2, Tnf, Hspd1, Ing5, Rassf2, Casp3, C1qbp, Bcl2a1d, Bcl2a1b, Tgm2, Utp11, Ripk2, Txnrd1, Rrp1b, Siah1b, Dhodh, Dnaja1, Nr4a1, Melk, Il1b, Lcn2,*
Steroid metabolic process (GO:0008202)	15	3.12E-04	0.014272545	*Fdps, Hmgcs1, Insig1, Dhcr24, Msmo1, Hmgcr, Srebf2, Cyp51, Nsdhl, Erg28, Chst10, Dhcr7, Ldlr, Lbr, Fdft1*
Cholesterol metabolic process (GO:0008203)	15	5.46E-05	0.003626313	*Fdps, Hmgcs1, Insig1, Lrp5, Dhcr24, Msmo1, Hmgcr, Srebf2, Cyp51, Sqle, Nsdhl, Dhcr7, Ldlr, Lbr, Fdft1*
Response to virus (GO:0009615)	13	1.38E-04	0.00748434	*Ivns1abp, Ifitm3, Ifitm2, Ddx3x, Rsad2, Ddx1, Odc1, Ddx21, Irak3, Tnf, Il17ra, Lcn2, Oasl1*
Steroid biosynthetic process (GO:0006694)	12	2.01E-05	0.001716264	*Fdps, Nsdhl, Erg28, Hmgcs1, Dhcr24, Msmo1, Dhcr7, Hmgcr, Hsd17b7, Lbr, Cyp51, Fdft1*
Cellular response to virus (GO:0098586)	11	1.38E-04	9.55E-04	*Cxcl10, Hsp90aa1, Ddx3x, Tomm70a, Fmr1, Zc3h12a, Gbf1, Rrp1b, Pou2f2, Ikbke, Nfkb1p*
Cellular response to oxidative stress (GO:0034599)	11	0.00106265	0.04040636	*Nr4a2, Chchd4, Pnpt1, Zc3h12a, G6pdx, Gsr, Slc11a2, Atp2a2, Pycr2, Sod2, Eif2s1*
Cellular response to interleukin-17 (GO:0097398)	5	0.00101988	0.039252839	*Cxcl10, Il1b, Srsf1, Nfkbiz, Nfkb1*
Cellular amino acid biosynthetic process (GO:0008652)	4	0.05200478	0.582592119	*Mthfd1, Pycr2, Bcat1, Enoph1*

One-sided *p*-values for Fisher's Exact test* and Benjamini–Hochberg correction for multiple comparisons to obtain *p*-adjusted**.

Supplementary Table 5. Biological processes enriched with downregulated genes upon NLRP3 p.D303N expression.

GO term	Gene count	*p*-value*	Benjamini–Hochberg**	Genes
Lipid metabolic process (GO:0006629)	42	3.1121E-05	0.011874445	*Dgkg, Faah, B4galt1, Cerk, Insig2, Mgst3, Hexb, Hexa, Inppl1, Lpl, Ptpn22, Hacd4, Ptgs1, Pld2, Sult1a1, Hmgcl, Gm2a, Psap, Cyp4v3, Scd2, Pgap6, Inpp5k, Hacl1, Pltp, Hsd3b7, Cpt1a, Gpx1, Sphk2, Sphk1, Nr1h2, Ephx1, Peds1, Plcb3, Soat1, Naaa, Acot2, Rubcnl, Echdc3, Acox3, Pafah1b3, Pam, Slc27a4*
Positive regulation of transcription (GO:0045893)	41	8.7396E-05	0.020332626	*Kdm3a, Calcoco1, Shc1, Cited2, Wbp2, Src, Pbxip1, Rora, Foxo3, Hif1a, Npas2, Phf8, Lbh, Tnni2, Inpp5k, Apbb1, Nos1, Mta3, Trp53inp1, Arhgef11, Arnt2, Egr2, Jun, Niban2, Map3k1, Fzd7, Nr1h2, Tfeb, Dyrk1b, Mitf, Klf4, Usf2, Runx3, Trerf1, Klf2, Mafb, Asph, Id2, Irf8, Mapre3, Map3k12*
Positive regulation of cell migration (GO:0030335)	25	9.0474E-07	6.21E-04	*Grn, Flt1, Cxcr4, Lamc2, Vsir, Gnai2, Pld2, Ppp3ca, Plau, Ccl3, Itgax, S1pr1, C3ar1, Ccr1, Sema4a, Sphk1, Cav1, Sema4g, Vegfa, Cpeb1, Myadm, Numb, Spry2, Myo1f, Bcar1*
Cell proliferation (GO:0008283)	25	9.4735E-05	0.020332626	*Rb1, Rarg, B4galt1, Cited2, Src, Adm, Rasgrp4, Gnai2, Ndst1, S1pr1, Mta3, Tns3, Appl2, Pdk1, Jun, Gpx1, Sphk2, Sphk1, Cav1, Vegfa, Asph, Kitl, Tgfbi, Ypel5, Nodal*
Response to hypoxia (GO:0001666)	23	1.3772E-07	1.58E-04	*Egln1, Arnt2, Camk2d, Cdkn1b, Flt1, Cited2, Cav1, Bnip3, Cxcr4, Adm, Plod1, Hif1a, Vegfa, Pld2, Cd24a, Plau, Ddit4, Acot2, Capn2, Lonp1, Hmox1, Nos1, Pam*
Positive regulation of ERK1, ERK2 cascade (GO:0070374)	20	7.5230E-05	0.020332626	*Ccr1, App, Camk2d, Jun, Shc1, Src, Cxcr4, Trem2, Arrb1, Ptpn22, Mturn, Vegfa, Gnai2, Prxl2c, Nptn, Ccl3, Spry2, Nodal, Map3k12, Mapk3*
Cellular response to hypoxia (GO:0071456)	18	1.2000E-07	1.58E-04	*Egln1, Bnip3l, Src, Bnip3, Trem2, Rora, Ak4, Foxo3, Ndrg1, Hif1a, Rtn4, Vegfa, Cpeb1, Pink1, Rgcc, Eif4ebp1, Hmox1, Mgarp*
Carbohydrate metabolic process (GO:0005975)	17	4.94E-04	0.072104731	*Epm2a, B4galt1, Gbe1, Hexb, Hexa, Pygl, Hexdc, Hk1, Gpi1, Hk3, Ppp1r3c, Hyal1, Ppp1r3b, Gapdh, Pdk2, Pgm1, Pdk1*
Positive regulation of angiogenesis (GO:0045766)	17	1.7689E-05	0.010124058	*Ecm1, Grn, Flt1, Sphk1, Emp2, Adm, Hif1a, Rtn4, Add1, Vegfa, Rhob, Lgals3, Hyal1, C3ar1, Itgax, Hmox1, Nodal*
Actin filament organization (GO:0007015)	16	8.3825E-05	0.020332626	*Tmod1, Gsn, Sh3kbp1, Inppl1, Emp2, Arhgap25, Arhgap6, Rnd3, Mtss1, Coro1b, Rhob, Myo1e, Whamm, Rhoq, Myo1f, Bcar1*

One-sided *p*-values for Fisher's Exact test* and Benjamini–Hochberg correction for multiple comparisons to obtain *p*-adjusted**.
