# Supplementary material for: Pathogenic NLRP3 mutants form constitutively active inflammasomes resulting in immune-metabolic limitation of IL-1β production
Source: Nat Commun. 2024 Feb 6;15:1096. doi: 10.1038/s41467-024-44990-0 (PMC10847128; doi:10.1038/s41467-024-44990-0)
Supplement: Supplementary file 3 — Description of Additional Supplementary Files [file 41467_2024_44990_MOESM3_ESM.pdf]

Description of Additional Supplementary Files

File Name: Supplementary Data 1
Description: A single Excel file including data of the compounds detected in metabolomic analysis of monocytes from healthy donors and CAPS patients.

File Name: Supplementary Data 2
Description: A single Excel file including data of the compounds detected in metabolomic analysis of immortalized macrophages.
