# Supplementary material for: Pathogenic NLRP3 mutants form constitutively active inflammasomes resulting in immune-metabolic limitation of IL-1β production
Source: Nat Commun. 2024 Feb 6;15:1096. doi: 10.1038/s41467-024-44990-0 (PMC10847128; doi:10.1038/s41467-024-44990-0)
Supplement: Supplementary file 6 — Reporting Summary [file 41467_2024_44990_MOESM6_ESM.pdf]

nature portfolio

Corresponding author(s): Pablo Pelegrin

Last updated by author(s): Dec 15, 2023

Reporting Summary

Nature Portfolio wishes to improve the reproducibility of the work that we publish. This form provides structure for consistency and transparency in reporting. For further information on Nature Portfolio policies, see our Editorial Policies and the Editorial Policy Checklist.

Statistics

For all statistical analyses, confirm that the following items are present in the figure legend, table legend, main text, or Methods section.

n/a	Confirmed	
☐	☒	The exact sample size (*n*) for each experimental group/condition, given as a discrete number and unit of measurement
☐	☒	A statement on whether measurements were taken from distinct samples or whether the same sample was measured repeatedly
☐	☒	The statistical test(s) used AND whether they are one- or two-sided<br>*Only common tests should be described solely by name; describe more complex techniques in the Methods section.*
☐	☒	A description of all covariates tested
☐	☒	A description of any assumptions or corrections, such as tests of normality and adjustment for multiple comparisons
☐	☒	A full description of the statistical parameters including central tendency (e.g. means) or other basic estimates (e.g. regression coefficient) AND variation (e.g. standard deviation) or associated estimates of uncertainty (e.g. confidence intervals)
☐	☒	For null hypothesis testing, the test statistic (e.g. *F*, *t*, *r*) with confidence intervals, effect sizes, degrees of freedom and *P* value noted<br>*Give P values as exact values whenever suitable.*
☒	☐	For Bayesian analysis, information on the choice of priors and Markov chain Monte Carlo settings
☒	☐	For hierarchical and complex designs, identification of the appropriate level for tests and full reporting of outcomes
☒	☐	Estimates of effect sizes (e.g. Cohen's *d*, Pearson's *r*), indicating how they were calculated

Our web collection on statistics for biologists contains articles on many of the points above.

Software and code

Policy information about availability of computer code

Data collection	Gene5 & Gene 5 Secure (Biotek Instruments, USA), Image Lab Touch Software (Biorad, USA), iQ5 Optical System software v2.0 (Biorad, USA), FACS diva software (BD, Bioscience), Imaging Software NIS-Elements v4.3 (Nikon, Japan), MZmine software (Version 2.53, MZmine Development Team), Wave software version 2.6 (Agilent Technologies), NextSeq 2000 Sequencing Systems (Illumina),MassHunter Qualitative Analysis software (Version 10.0, Agilent Technologies)
Data analysis	Prism (GraphPad Inc., v9), FCS Express 4 Flow Research (De novo software), ImageJ (US National Institutes of Health, Bethesda, MD USA), Gene5 & Gene 5 Secure (Biotek Instruments, USA), Limma (R studio, Posit, PBC), DESeq2 (R studio, Posit, PBC), Metaboanalyst 5.0 online platform (www.metaboanalyst.ca), Mass Profiler professional (MPP, Agilent technologies), DAVID tool v2021.

For manuscripts utilizing custom algorithms or software that are central to the research but not yet described in published literature, software must be made available to editors and reviewers. We strongly encourage code deposition in a community repository (e.g. GitHub). See the Nature Portfolio guidelines for submitting code & software for further information.

Data

Policy information about availability of data

All manuscripts must include a data availability statement. This statement should provide the following information, where applicable:

- Accession codes, unique identifiers, or web links for publicly available datasets
- A description of any restrictions on data availability
- For clinical datasets or third party data, please ensure that the statement adheres to our policy

The metabolomic data generated in this study have been deposited in the MetaboLights database under accession code MTBLS7872 [https://www.ebi.ac.uk/metabolights/MTBLS7872]. The RNAseq data generated in this study have been deposited in the GEO database under accession code GSE246713 [https://www.ncbi.nlm.nih.gov/geo/query/acc.cgi?acc=GSE246713]. The RNAseq data used in this study are available in the GEO database under accession codes GSE57253, GSE42606 and GSE17732 [https://www.ncbi.nlm.nih.gov/geo/query/acc.cgi?acc=GSE57253; https://www.ncbi.nlm.nih.gov/geo/query/acc.cgi?acc=GSE42606; https://www.ncbi.nlm.nih.gov/geo/query/acc.cgi?acc=GSE17732]. Source data are provided with this paper.

Research involving human participants, their data, or biological material

Policy information about studies with human participants or human data. See also policy information about sex, gender (identity/presentation), and sexual orientation and race, ethnicity and racism.

Reporting on sex and gender	Sex of participants is annotated in Table S1. Since CAPS are rare disease, we could not take sex in study design as we had access to few CAPS patients. Gender analysis was not included in the informed consent.
Reporting on race, ethnicity, or other socially relevant groupings	Race and ethnicity analysis was not included in the informed consent and was not collected.
Population characteristics	Age, sex, mutation, diagnostic and treatment of CAPS patients as well as healthy population is presented in Table S1.
Recruitment	Inclusion criteria for patient recruitment were: (1) age over 18 years; (2) CAPS diagnostic; (3) confirmed pathogenic mutation in NLRP3 gene; (4) signed informed consent. The particular NLRP3 muation carrying the patients, the clinical phenotype of the CAPS (MWS and MWS/CINCA) as well as current medication (Canakinumab and Anakinra) might bias the results.
Ethics oversight	Approval of the Ethical Committee of the Clinical University Hospital Virgen de la Arrixaca (Murcia, Spain)

Note that full information on the approval of the study protocol must also be provided in the manuscript.

Field-specific reporting

Please select the one below that is the best fit for your research. If you are not sure, read the appropriate sections before making your selection.

☒ Life sciences ☐ Behavioural & social sciences ☐ Ecological, evolutionary & environmental sciences

For a reference copy of the document with all sections, see nature.com/documents/nr-reporting-summary-flat.pdf

Life sciences study design

All studies must disclose on these points even when the disclosure is negative.

Sample size	The sample size for human samples was determined based on prior studies that reported ex vivo experiments with CAPS samples (Tapia-Abellán et al., Nat Chem Biol 2019). Given that CAPS is a rare disease with a limited patient population, we also faced constraints in terms of available resources.
Data exclusions	Outliers from data sets were identified by the ROUT method with Q=1% using Prism v9 software (GraphPad Inc).
Replication	Experiments were repeated at least three times and/or with sufficient cells per group to demonstrate statistical significance. All replication experiments give a similar result as the presented representative in main figures. In the case of human samples, due to the limitation of the samples and the cell numbers, the experiment from each patient or donor was done once for each patient.
Randomization	Randomization is not considered relevant for the in vitro assays presented in this study, as the risk of bias is already minimized by the controlled environment where the experiments were performed. We declare that the experiments were conducted with the highest level of rigor and transparency following reported methods.
Blinding	Blinding is not considered relevant for the in vitro assays presented in this study, as the risk of bias is already minimized by the controlled environment where the experiments were performed. We declare that the experiments were conducted with the highest level of rigor and transparency following reported methods.

Reporting for specific materials, systems and methods

We require information from authors about some types of materials, experimental systems and methods used in many studies. Here, indicate whether each material, system or method listed is relevant to your study. If you are not sure if a list item applies to your research, read the appropriate section before selecting a response.

Materials & experimental systems

n/a	Involved in the study
☐	☒ Antibodies
☐	☒ Eukaryotic cell lines
☒	☐ Palaeontology and archaeology
☒	☐ Animals and other organisms
☐	☒ Clinical data
☐	☒ Dual use research of concern
☒	☐ Plants

Methods

n/a	Involved in the study
☒	☐ ChIP-seq
☐	☒ Flow cytometry
☒	☐ MRI-based neuroimaging

Antibodies

Antibodies used

PE conjugated mouse monoclonal anti-ASC antibody (clone HASC-71, catalogue 653903, Biolegend, 1:500); FITC conjugated mouse monoclonal anti-CD14 antibody (clone M5E2, catalogue 557153, BD Biosciences, 1:10); PE-Cy7 conjugated mouse monoclonal anti-CD16 antibody (clone 3G8, catalogue 557744, BD Biosciences 1:10); rabbit polyclonal antibody anti-ASC (N-15)-R (sc-22514-R, Santa Cruz, 1:500); donkey anti-rabbit alexa-647 antibody (1:800, A31573, Life Technologies); anti-NLRP3 mouse monoclonal (Cryo-2 clone, AG-20B-0014, Adipogen, 1:1000), anti-Caspase 1 (p20) mouse monoclonal (Casper-1, AG-20B-0042, Adipogen, 1:1000), anti-GSDMD rabbit monoclonal (EPR19828, ab209845, Abcam, 1:2500), anti-IL-1β rabbit polyclonal (H-153; sc-7884, 1:1000); horseradish peroxidase (HRP)-anti-β-actin (C4; sc-47778HRP, Santa Cruz, 1:10,000); horseradish peroxidase anti-IgG rabbit (NA9340V, Cytiva, 1:5000) and horseradish peroxidase anti-IgG mouse (NA9341V, Cytiva, 1:5000).

Validation

All antibodies used came from commercial vendors, and we based specificity on their provided description and data sheets.
1.anti-ASC antibody (clone HASC-71, catalogue 653903), Biolegend) ; RRID:AB_2564507
https://www.labome.com/product/BioLegend/653903.html
Scambler T, Jarosz Griffiths H, Lara Reyna S, Pathak S, Wong C, Holbrook J, et al. ENaC-mediated sodium influx exacerbates NLRP3-dependent inflammation in cystic fibrosis. elife. 2019;8
2. anti-CD14 antibody (clone M5E2, catalogue 557153, BD Biosciences); RRID:AB_396589
https://www.bdbiosciences.com/en-us/products/reagents/flow-cytometry-reagents/research-reagents/single-color-antibodies-ruo/fitc-mouse-anti-human-cd14.557153
Malhotra, S., Hurtado-Navarro, L., Pappolla, A., Villar, L. M., Río, J., Montalban, X., ... & Comabella, M. (2023). Increased nlrp3 inflammasome activation and pyroptosis in patients with multiple sclerosis with fingolimod treatment failure. Neurology® Neuroimmunology & Neuroinflammation, 10(3).
3. anti-CD16 antibody (clone 3G8, catalogue 557744, BD Biosciences); RRID:AB_396850
https://www.bdbiosciences.com/en-de/products/reagents/flow-cytometry-reagents/research-reagents/single-color-antibodies-ruo/pe-cy-7-mouse-anti-human-cd16.557744
Malhotra, S., Hurtado-Navarro, L., Pappolla, A., Villar, L. M., Río, J., Montalban, X., ... & Comabella, M. (2023). Increased nlrp3 inflammasome activation and pyroptosis in patients with multiple sclerosis with fingolimod treatment failure. Neurology® Neuroimmunology & Neuroinflammation, 10(3).
4. anti-ASC (N-15)-R (sc-22514-R, Santa Cruz); RRID:AB_2174874
https://www.scbt.com/es/p/asc-antibody-n-15
Akula, M. K., Shi, M., Jiang, Z., Foster, C. E., Miao, D., Li, A. S., ... & Wang, D. (2016). Control of the innate immune response by the mevalonate pathway. Nature immunology, 17(8), 922-929.
5. anti-NLRP3 mouse (Cryo-2 clone, AG-20B-0014, Adipogen); RRID:AB_2490202
https://adipogen.com/ag-20b-0014-anti-nlrp3-nalp3-mab-cryo-2.html/
Baroja-Mazo, A., Martín-Sánchez, F., Gomez, A. I., Martínez, C. M., Amores-Iniesta, J., Compan, V., ... & Pelegrín, P. (2014). The NLRP3 inflammasome is released as a particulate danger signal that amplifies the inflammatory response. Nature immunology, 15(8), 738-748.
6. anti-Caspase 1 (p20) mouse (Casper-1, AG-20B-0042, Adipogen); RRID:AB_2490248
https://adipogen.com/ag-20b-0042-anti-caspase-1-p20-mouse-mab-casper-1.html
Baroja-Mazo, A., Martín-Sánchez, F., Gomez, A. I., Martínez, C. M., Amores-Iniesta, J., Compan, V., ... & Pelegrín, P. (2014). The NLRP3 inflammasome is released as a particulate danger signal that amplifies the inflammatory response. Nature immunology, 15(8), 738-748.
7. anti-GSDMD rabbit (EPR19828, ab209845, Abcam); RRID:AB_2783550
https://www.abcam.com/products/primary-antibodies/gsdmd-antibody-epr19828-ab209845.html
ZZhang LM et al. STING mediates neuroinflammatory response by activating NLRP3-related pyroptosis in severe traumatic brain injury. J Neurochem 162:444-462 (2022).
8. anti-IL-1β rabbit (H-153; sc-7884, Santa Cruz); RRID:AB_2124476
https://www.scbt.com/es/p/il-1beta-antibody-h-153
Baroja-Mazo, A., Martín-Sánchez, F., Gomez, A. I., Martínez, C. M., Amores-Iniesta, J., Compan, V., ... & Pelegrín, P. (2014). The NLRP3 inflammasome is released as a particulate danger signal that amplifies the inflammatory response. Nature immunology, 15(8), 738-748.

Eukaryotic cell lines

Policy information about cell lines and Sex and Gender in Research

Cell line source(s)	HEK293T cells (CRL-11268; American Type Culture Collection), Immortalized mouse bone marrow macrophages (Nat Commun. 2018;9:5182); RAW 264.7 macrophages expressing a reporter SEAP gen for NF-kB (raw-sp, Invivogen)
Authentication	None of the cell lines used were authenticated
Mycoplasma contamination	HEK293T cell line were routinely tested for Mycoplasma contamination and were negative for Mycoplasma. Immortalized mouse bone marrow macrophages and RAW 264.7 macrophages expressing a reporter SEAP gen for NF-kB were not routinely tested for Mycoplasma.
Commonly misidentified lines (See ICLAC register)	No misidentified cell lines were used in the study.

Clinical data

Policy information about clinical studies

All manuscripts should comply with the ICMJE guidelines for publication of clinical research and a completed CONSORT checklist must be included with all submissions.

Clinical trial registration	N/A
Study protocol	N/A
Data collection	N/A
Outcomes	N/A

Flow Cytometry

Plots

Confirm that:

☒ The axis labels state the marker and fluorochrome used (e.g. CD4-FITC).

☒ The axis scales are clearly visible. Include numbers along axes only for bottom left plot of group (a 'group' is an analysis of identical markers).

☒ All plots are contour plots with outliers or pseudocolor plots.

☒ A numerical value for number of cells or percentage (with statistics) is provided.

Methodology

Sample preparation	Intracellular ASC-speck formation in human monocytes was evaluated by seeding 50 µl of individuals' whole blood samples in polystyrene flow cytometry tubes (Falcon) with RPMI 1640 medium (Lonza) containing 10% FCS and 2mM Glutamax. Following treatments with inhibitor or triggers, cells were stained for the detection of ASC specks by Time-of-Flight Inflammasome Evaluation (TOFIE) 59,60 using the PE conjugated mouse monoclonal anti-ASC antibody (clone HASC-71, catalogue 653903, Biolegend, 1:500). Monocytes were gated using the FITC conjugated mouse monoclonal anti-CD14 antibody (clone M5E2, catalogue 557153, BD Biosciences, 1:10) and using the PE-Cy7 conjugated mouse monoclonal anti-CD16 antibody (clone 3G8, catalogue 557744, BD Biosciences 1:10).<br><br>Intracellular ASC-RFP-speck formation in HEK293T cells was evaluated after 24 h post-transfection by TOFIE in different gates with increasing mean fluorescence intensity for NLRP3-YFP
Instrument	FACS Canto cytometer (BD Biosciences)
Software	FACS diva software (BD Biosciences) & FCS Express 4 Flow Research (De novo software)
Cell population abundance	Monocytes were the population of interest and accounted between 15-20% of PBMCs after Ficoll gradient isolation from whole blood.
Gating strategy	For ASC specking monocytes, monocytes were gated from blood cells using CD14-FITC and CD16-PE-Cy7, and then ASC specking monocytes were gated in a ASC-PE-Width vs ASC-PE-Area dot plot.<br><br>For HEK293T they were gated in dot plot NLRP3-YFP and ASC-RFP.

☒ Tick this box to confirm that a figure exemplifying the gating strategy is provided in the Supplementary Information.
